# Supplementary material for: CRB2 Loss in Rod Photoreceptors Is Associated with Progressive Loss of Retinal Contrast Sensitivity
Source: Int J Mol Sci. 2019 Aug 21;20(17):4069. doi: 10.3390/ijms20174069 (PMC6747345; doi:10.3390/ijms20174069)
Supplement: Supplementary file 1 [file ijms-20-04069-s001.pdf]

Supplementary Figure S1. Specific ablation of *Crb2* from rod photoreceptors. Evaluation of the recombination efficiency of a Rho-*iCre* mouse line (A-F). The R26-stop-*EYFP* mutant mice have a loxP-flanked STOP sequence followed by the Enhanced Yellow Fluorescent Protein gene (*EYFP*) inserted into the Gt(ROSA)26Sor locus ($EYFP^{flox\text{-}stop\text{-}flox/+}$). When bred to mice expressing Rho-*iCre*, the STOP sequence is deleted and EYFP expression is observed in the rod photoreceptor cells of the double mutant offspring ($Crb2^{\Delta Rods}$::*EYFP*). Confocal laser scanning microscope pictures of (postnatal day 20 (P20)) retina sections from $Crb2^{flox/flox}$::$EYFP^{flox\text{-}stop\text{-}flox/+}$ (A-C), and $Crb2^{\Delta Rods}$::*EYFP* (D-F), stained with anti-GFP and anti-cone arrestin (CAR), and sections from $Crb2^{flox/flox}$::$EYFP^{flox\text{-}stop\text{-}flox/+}$ (G), and $Crb2^{\Delta Rods}$::*EYFP* (H), stained with anti-CRB2 antibodies. While in the $Crb2^{flox/flox}$::$EYFP^{flox\text{-}stop\text{-}flox/+}$ (A and C) no EYFP signal could be detected, in $Crb2^{\Delta Rods}$::*EYFP* (F, arrows) retinas expression of EYFP was found only in the outer nuclear and inner-segment layers. No co-localization of EYFP with cone arrestin positive cells was observed (D, E, F, arrows), suggesting that recombination was restricted to rod photoreceptor cells. CRB2 protein was detected at the subapical region in the control retina (G). Besides the ablation of *Crb2* from rods photoreceptors, the CRB2 protein could still be detected at the subapical region of mutant retinas once the protein is still expressed by wild-type Müller glial cells and cone photoreceptors (H). CAR, cone arrestin; INL, inner nuclear layer; ONL, outer nuclear layer; OLM, outer limiting membrane. Scale bar: (A-H): 25 µm.

Supplementary Figure S2. $Crb1^{KO}Crb2^{flox/flox}$ and $Crb2^{flox/flox}$ mice present similar scotopic electroretinography responses. Electroretinographic analysis of retinal function in $Crb1^{KO}Crb2^{flox/flox}$ (black) and $Crb2^{flox/flox}$ (gray) at different time points, 1-, 3-, 6-, 9-, and 12-months-of-age. Boxes indicate the 25 and 75% quantile range and whiskers indicate the 5 and 95% quantiles, the intersection of line and error bar indicates the median of the data (box-and-whisker plot).

Supplementary Figure S3. $Crb2^{\Delta Rods}$ and $Crb1^{KO}Crb2^{\Delta Rods}$ mice present normal photopic electroretinography responses. Electroretinographic analysis of retinal function in $Crb2^{flox/flox}$ (control) (gray), $Crb2^{\Delta Rods}$ (blue) and $Crb1^{KO}Crb2^{\Delta Rods}$ (red) at different time points, 1-, 3-, 6-, 9- and 12-months-of-age. Boxes indicate the 25 and 75% quantile range and whiskers indicate the 5 and 95% quantiles, and the intersection of line and error bar indicates the median of the data (box-and-whisker plot).

Supplementary Figure S4. Similar contrast sensitivity for $Crb1^{KO}Crb2^{flox/flox}$ and $Crb2^{flox/flox}$ mice at all frequencies measured. (A) Contrast sensitivity in cycles per degree (c/d) from 1-month of age (1M), 3M, 7M and 9M $Crb1^{KO}Crb2^{flox/flox}$. No difference was detected between $Crb1^{KO}Crb2^{flox/flox}$ mice at all frequencies and time points measured. (B) Contrast sensitivity from 5-month of age $Crb2^{flox/flox}$ and $Crb2^{\Delta Rods}$. No difference was observed between $Crb2^{flox/flox}$ and $Crb2^{\Delta Rods}$ mice. Error bars indicate ± SEM.

Supplementary Figure S5. Schematic overview of retinal degeneration. (A) Loss of CRB2 from immature rod and cone photoreceptors results at 5M in severe disruption of adherens junctions and nearly complete loss of photoreceptor cells throughout the entire retina (Alves et al 2014). (B) loss of CRB2 in mature rod photoreceptors results at 9M in milder disruption of adherens junctions and photoreceptor loss especially at the far periphery especially of the superior retina (current data) The loss of CRB2 in rod photoreceptors also results at 9M in gliosis (GFAP upregulation) in the adjacent Müller glial cells.
